# Diethyl 5-[(2-hydr­oxy-1-naphth­yl)methyl­ideneamino]-3-methyl­thio­phene-2,4-dicarboxyl­ate

**DOI:** 10.1107/S1600536808011628

**Published:** 2008-04-26

**Authors:** Mehmet Akkurt, Şerife Pınar Yalçın, Abdullah Mohamed Asiri, Orhan Büyükgüngör

**Affiliations:** a Department of Physics, Faculty of Arts and Sciences, Erciyes University, 38039 Kayseri, Turkey; b Chemistry Department, Faculty of Science, King Abdul-Aziz University, PO Box 80203, Jeddah 21589, Saudi Arabia; c Department of Physics, Faculty of Arts and Sciences, Ondokuz Mayıs University, 55139 Samsun, Turkey

## Abstract

In the title compound, C_22_H_21_NO_5_S, the 2-naphthol group and the thio­phene ring are almost coplanar, with a dihedral angle of 5.75 (7)°. The structure is stabilized by intra­molecular O—H⋯O, O—H⋯N and C—H⋯S, and inter­molecular C—H⋯O hydrogen-bonding inter­actions.

## Related literature

For related structures, see: Akkurt, Karaca *et al.* (2008 ▶); Akkurt, Yıldırım *et al.* (2008 ▶); Asiri & Badahdah (2007 ▶). For bond-length data, see: Allen *et al.* (1987 ▶).
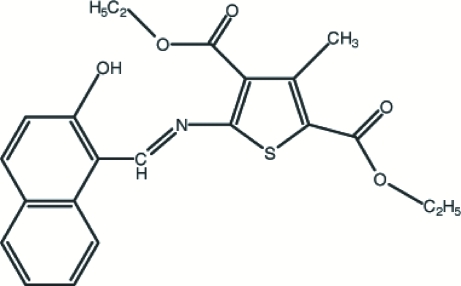


## Experimental

### 

#### Crystal data

C_22_H_21_NO_5_S
*M* *_r_* = 411.47
Triclinic, 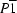

*a* = 8.7111 (4) Å
*b* = 11.5319 (5) Å
*c* = 11.9778 (5) Å
α = 61.594 (3)°
β = 79.489 (3)°
γ = 89.334 (3)°
*V* = 1036.67 (9) Å^3^
*Z* = 2
Mo *K*α radiation
μ = 0.19 mm^−1^
*T* = 293 (2) K
0.63 × 0.38 × 0.10 mm

#### Data collection

Stoe IPDS-2 diffractometer
Absorption correction: integration (*X-RED32*; Stoe & Cie) *T*_min_ = 0.890, *T*_max_ = 0.981
18909 measured reflections
4047 independent reflections
3397 reflections with *I* > 2σ(*I*)
*R*_int_ = 0.029

#### Refinement

*R*[*F* ^2^ > 2σ(*F* ^2^)] = 0.040
*wR*(*F* ^2^) = 0.111
*S* = 1.06
4047 reflections
265 parameters
H-atom parameters constrained
Δρ_max_ = 0.29 e Å^−3^
Δρ_min_ = −0.15 e Å^−3^

### 

Data collection: *X-AREA* (Stoe & Cie, 2002 ▶); cell refinement: *X-AREA*; data reduction: *X-RED32*; program(s) used to solve structure: *SHELXS97* (Sheldrick, 2008 ▶); program(s) used to refine structure: *SHELXL97* (Sheldrick, 2008 ▶); molecular graphics: *ORTEP-3 for Windows* (Farrugia, 1997 ▶); software used to prepare material for publication: *WinGX* (Farrugia, 1999 ▶).

## Supplementary Material

Crystal structure: contains datablocks global, I. DOI: 10.1107/S1600536808011628/sj2483sup1.cif

Structure factors: contains datablocks I. DOI: 10.1107/S1600536808011628/sj2483Isup2.hkl

Additional supplementary materials:  crystallographic information; 3D view; checkCIF report

## Figures and Tables

**Table 1:** Hydrogen-bond geometry (Å, °)

*D*—H⋯*A*	*D*—H	H⋯*A*	*D*⋯*A*	*D*—H⋯*A*
O1—H1*A*⋯O3	0.82	2.58	3.209 (2)	135
O1—H1*A*⋯N1	0.82	1.83	2.561 (2)	147
C1—H1⋯S1	0.93	2.59	3.0263 (18)	109
C7—H7⋯O4^i^	0.93	2.37	3.269 (3)	163
C16—H16*C*⋯O4	0.96	2.25	2.978 (3)	132
C21—H21*B*⋯O2^ii^	0.97	2.60	3.565 (3)	175

Symmetry codes: (i) 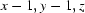; (ii) 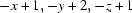.

**Fig. 1.**
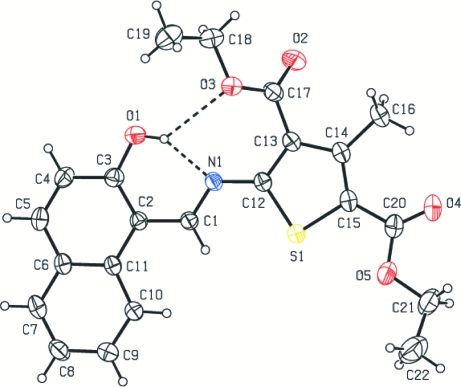
A view of the title compound, I, with the atom-numbering scheme, and 30% probability displacement ellipsoids. Classical intramolecular H-bonds are drawn as dashed lines.

**Fig. 2.**
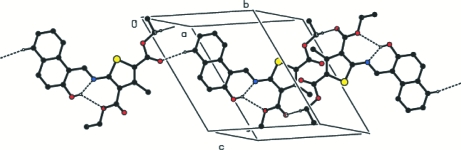
The crystal packing of I with hydrogen bonds drawn as dashed lines. H atoms not involved in hydrogen bonding have been omitted.

## supplementary crystallographic information

### Comment

We recently reported the structures of 4-[(2-hydroxy-1-naphthyl)methylideneamino]benzoic acid (Akkurt, Yıldırım *et al.*, 2008) and 2-[(2-Hydroxybenzylidene) amino]-3-methoxycarbonyl-3,4,5,6-tetrahydrobenzo[*d*] thiophene (Akkurt, Karaca *et al.*, 2008). In this communication, we report the structure of the title compound, 4-[(2-hydroxynaphth-1-yl methylidene) amino]-3,5-diethoxycarbonyl- 4-methylthiophene (I), as a part of an ongoing investigation into the development of anil derivatives.

In the title compound (Fig. 1), all  values of the geometric parameters are normal (Allen *et al.*, 1987). The naphthalen-2-ol group and thiophene ring are each almost planar, with maximum deviations of -0.020 (1) Å for O1 and 0.008 (2) Å for C15, respectively, and the respective ring planes are oriented with a dihedral angle of 5.75 (7)° between them. Intramolecular O1—H1A···O3,  O1—H1A···N1 and C1—H1···S hydrogen bonds effect this conformation and the structure is further stabilised by intermolecular C—H···O hydrogen bonding interactions (Table 1 and Fig. 2).

### Experimental

The title compound, I, was prepared by the method of  Asiri & Badahdah (2007) and recrystallised from ethanol.  [Yield 99%, mp: 448 K].

### Refinement

The H atoms were positioned geometrically (C—H = 0.93 - 0.97 Å, O—H = 0.82 Å) and refined as riding with with *U*_iso_(H) = 1.2*U*_eq_(C_aromatic_, C_methylene_) and 1.5*U*_eq_(C_methyl_,*O*_hydroxyl_).

### Crystal data

**Table:**

C_22_H_21_NO_5_S	*Z* = 2
*M**_r_* = 411.47	*F*_000_ = 432
Triclinic, *P*1	*D*_x_ = 1.318 Mg m^−^^3^
Hall symbol: -P 1	Mo *K*α radiation λ = 0.71073 Å
*a* = 8.7111 (4) Å	Cell parameters from 18909 reflections
*b* = 11.5319 (5) Å	θ = 2.0–28.0º
*c* = 11.9778 (5) Å	µ = 0.19 mm^−^^1^
α = 61.594 (3)º	*T* = 293 (2) K
β = 79.489 (3)º	Plate, orange
γ = 89.334 (3)º	0.63 × 0.38 × 0.10 mm
*V* = 1036.67 (9) Å^3^	

### Data collection

**Table:**

Stoe IPDS-2 diffractometer	4047 independent reflections
Monochromator: plane graphite	3397 reflections with *I* > 2σ(*I*)
Detector resolution: 6.67 pixels mm^-1^	*R*_int_ = 0.029
*T* = 293(2) K	θ_max_ = 26.0º
ω scans	θ_min_ = 2.0º
Absorption correction: integration(X-RED32; Stoe & Cie)	*h* = −10→10
*T*_min_ = 0.890, *T*_max_ = 0.981	*k* = −14→14
18909 measured reflections	*l* = −14→14

### Refinement

**Table:**

Refinement on *F*^2^	Secondary atom site location: difference Fourier map
Least-squares matrix: full	Hydrogen site location: inferred from neighbouring sites
*R*[*F*^2^ > 2σ(*F*^2^)] = 0.040	H-atom parameters constrained
*wR*(*F*^2^) = 0.111	*w* = 1/[σ^2^(*F*_o_^2^) + (0.064*P*)^2^ + 0.1098*P*] where *P* = (*F*_o_^2^ + 2*F*_c_^2^)/3
*S* = 1.06	(Δ/σ)_max_ < 0.001
4047 reflections	Δρ_max_ = 0.29 e Å^−^^3^
265 parameters	Δρ_min_ = −0.15 e Å^−^^3^
Primary atom site location: structure-invariant direct methods	Extinction correction: none

### Special details

**Table:**

Geometry. Bond distances, angles *etc*. have been calculated using the rounded fractional coordinates. All su's are estimated from the variances of the (full) variance-covariance matrix. The cell e.s.d.'s are taken into account in the estimation of distances, angles and torsion angles
Refinement. Refinement on *F*^2^ for ALL reflections except those flagged by the user for potential systematic errors. Weighted *R*-factors *wR* and all goodnesses of fit *S* are based on *F*^2^, conventional *R*-factors *R* are based on *F*, with *F* set to zero for negative *F*^2^. The observed criterion of *F*^2^ > σ(*F*^2^) is used only for calculating -*R*-factor-obs *etc*. and is not relevant to the choice of reflections for refinement. *R*-factors based on *F*^2^ are statistically about twice as large as those based on *F*, and *R*-factors based on ALL data will be even larger.

### Fractional atomic coordinates and isotropic or equivalent isotropic displacement parameters (Å^2^)

**Table:**

	*x*	*y*	*z*	*U*_iso_*/*U*_eq_	
S1	0.49573 (5)	0.76909 (4)	0.39643 (4)	0.0501 (1)	
O1	0.14230 (15)	0.32467 (13)	0.72848 (10)	0.0600 (4)	
O2	0.3809 (2)	0.60637 (15)	0.86430 (13)	0.0796 (6)	
O3	0.40230 (17)	0.44308 (13)	0.81405 (11)	0.0629 (4)	
O4	0.7590 (2)	1.03239 (15)	0.40759 (16)	0.0874 (6)	
O5	0.70559 (18)	0.99593 (13)	0.25144 (13)	0.0721 (5)	
N1	0.31832 (15)	0.53737 (13)	0.57083 (12)	0.0454 (4)	
C1	0.27937 (17)	0.52740 (15)	0.47657 (14)	0.0433 (4)	
C2	0.17178 (17)	0.42282 (15)	0.49800 (14)	0.0421 (4)	
C3	0.10639 (19)	0.32652 (16)	0.62436 (15)	0.0488 (5)	
C4	−0.0033 (2)	0.22477 (18)	0.64652 (17)	0.0576 (5)	
C5	−0.0463 (2)	0.21932 (18)	0.54624 (18)	0.0576 (5)	
C6	0.01516 (18)	0.31457 (16)	0.41623 (16)	0.0485 (5)	
C7	−0.0334 (2)	0.30932 (19)	0.31243 (19)	0.0599 (6)	
C8	0.0266 (2)	0.4000 (2)	0.18768 (19)	0.0648 (7)	
C9	0.1389 (2)	0.4991 (2)	0.16108 (17)	0.0613 (6)	
C10	0.1874 (2)	0.50831 (17)	0.25911 (15)	0.0517 (5)	
C11	0.12654 (17)	0.41718 (15)	0.39031 (14)	0.0431 (4)	
C12	0.41757 (18)	0.64162 (15)	0.54888 (14)	0.0440 (5)	
C13	0.46275 (18)	0.66050 (16)	0.64435 (15)	0.0460 (5)	
C14	0.56177 (19)	0.77890 (16)	0.59411 (16)	0.0491 (5)	
C15	0.59028 (19)	0.84540 (16)	0.46218 (17)	0.0504 (5)	
C16	0.6309 (2)	0.8209 (2)	0.6770 (2)	0.0646 (7)	
C17	0.4107 (2)	0.56996 (18)	0.78467 (16)	0.0545 (6)	
C18	0.3541 (4)	0.3472 (2)	0.9500 (2)	0.0952 (9)	
C19	0.3661 (5)	0.2151 (3)	0.9659 (3)	0.1222 (16)	
C20	0.6929 (2)	0.96689 (18)	0.37492 (19)	0.0595 (6)	
C21	0.8094 (3)	1.1108 (2)	0.1560 (2)	0.0924 (9)	
C22	0.8308 (5)	1.1111 (3)	0.0332 (3)	0.1284 (13)	
H1	0.32280	0.59040	0.39190	0.0520*	
H1A	0.20650	0.38710	0.70570	0.0900*	
H4	−0.04630	0.16100	0.73100	0.0690*	
H5	−0.11820	0.15120	0.56310	0.0690*	
H7	−0.10780	0.24260	0.32990	0.0720*	
H8	−0.00720	0.39580	0.12030	0.0780*	
H9	0.18160	0.56000	0.07550	0.0730*	
H10	0.26200	0.57600	0.23900	0.0620*	
H16A	0.67860	0.74820	0.73720	0.0970*	
H16B	0.54950	0.84730	0.72380	0.0970*	
H16C	0.70860	0.89400	0.62290	0.0970*	
H18A	0.42060	0.36170	0.99980	0.1140*	
H18B	0.24680	0.35730	0.98170	0.1140*	
H19A	0.33970	0.15150	1.05640	0.1830*	
H19B	0.47130	0.20690	0.93080	0.1830*	
H19C	0.29510	0.19930	0.92100	0.1830*	
H21A	0.90960	1.10730	0.18150	0.1110*	
H21B	0.76440	1.19080	0.14860	0.1110*	
H22A	0.91040	1.17940	−0.02880	0.1920*	
H22B	0.73400	1.12730	0.00280	0.1920*	
H22C	0.86210	1.02680	0.04420	0.1920*	

### Atomic displacement parameters (Å^2^)

**Table:**

	*U*^11^	*U*^22^	*U*^33^	*U*^12^	*U*^13^	*U*^23^
S1	0.0542 (2)	0.0489 (2)	0.0479 (2)	−0.0031 (2)	−0.0107 (2)	−0.0236 (2)
O1	0.0637 (8)	0.0669 (8)	0.0435 (6)	−0.0065 (6)	−0.0077 (5)	−0.0228 (5)
O2	0.1077 (12)	0.0840 (10)	0.0569 (7)	−0.0006 (9)	−0.0050 (7)	−0.0456 (7)
O3	0.0824 (9)	0.0577 (7)	0.0464 (6)	−0.0033 (6)	−0.0131 (6)	−0.0232 (5)
O4	0.1040 (12)	0.0666 (9)	0.0921 (11)	−0.0283 (8)	−0.0127 (9)	−0.0402 (8)
O5	0.0825 (10)	0.0542 (7)	0.0677 (8)	−0.0158 (7)	−0.0004 (7)	−0.0246 (6)
N1	0.0443 (7)	0.0495 (7)	0.0475 (7)	0.0003 (6)	−0.0099 (5)	−0.0270 (6)
C1	0.0424 (8)	0.0458 (8)	0.0438 (7)	0.0014 (6)	−0.0079 (6)	−0.0234 (6)
C2	0.0380 (7)	0.0451 (8)	0.0457 (7)	0.0024 (6)	−0.0078 (6)	−0.0240 (6)
C3	0.0447 (8)	0.0520 (9)	0.0481 (8)	0.0033 (7)	−0.0074 (6)	−0.0236 (7)
C4	0.0533 (10)	0.0517 (9)	0.0542 (9)	−0.0063 (7)	−0.0023 (7)	−0.0174 (8)
C5	0.0476 (9)	0.0517 (9)	0.0710 (10)	−0.0073 (7)	−0.0068 (8)	−0.0291 (8)
C6	0.0413 (8)	0.0500 (9)	0.0604 (9)	0.0019 (7)	−0.0104 (7)	−0.0313 (8)
C7	0.0524 (10)	0.0669 (11)	0.0770 (12)	−0.0012 (8)	−0.0186 (8)	−0.0459 (10)
C8	0.0705 (12)	0.0781 (13)	0.0631 (11)	0.0009 (10)	−0.0218 (9)	−0.0447 (10)
C9	0.0693 (12)	0.0675 (11)	0.0502 (9)	−0.0004 (9)	−0.0131 (8)	−0.0303 (8)
C10	0.0546 (9)	0.0548 (9)	0.0484 (8)	−0.0041 (7)	−0.0087 (7)	−0.0273 (7)
C11	0.0382 (7)	0.0459 (8)	0.0514 (8)	0.0037 (6)	−0.0099 (6)	−0.0281 (7)
C12	0.0408 (8)	0.0466 (8)	0.0491 (8)	0.0033 (6)	−0.0097 (6)	−0.0265 (7)
C13	0.0434 (8)	0.0513 (8)	0.0511 (8)	0.0048 (7)	−0.0107 (6)	−0.0305 (7)
C14	0.0470 (8)	0.0513 (9)	0.0608 (9)	0.0061 (7)	−0.0149 (7)	−0.0350 (8)
C15	0.0479 (9)	0.0466 (8)	0.0643 (10)	0.0032 (7)	−0.0131 (7)	−0.0321 (8)
C16	0.0669 (11)	0.0694 (12)	0.0754 (12)	−0.0004 (9)	−0.0208 (9)	−0.0468 (10)
C17	0.0555 (10)	0.0647 (11)	0.0516 (9)	0.0015 (8)	−0.0124 (7)	−0.0340 (8)
C18	0.144 (2)	0.0779 (15)	0.0481 (10)	−0.0184 (15)	−0.0121 (12)	−0.0198 (10)
C19	0.186 (4)	0.0726 (17)	0.0824 (17)	−0.0039 (19)	−0.0425 (19)	−0.0114 (13)
C20	0.0577 (10)	0.0493 (9)	0.0721 (11)	0.0000 (8)	−0.0081 (8)	−0.0314 (9)
C21	0.110 (2)	0.0604 (13)	0.0830 (15)	−0.0210 (13)	0.0127 (13)	−0.0261 (11)
C22	0.163 (3)	0.099 (2)	0.0858 (18)	−0.027 (2)	0.0238 (19)	−0.0308 (16)

### Geometric parameters (Å, °)

**Table:**

S1—C12	1.7247 (16)	C13—C14	1.424 (3)
S1—C15	1.728 (2)	C14—C15	1.362 (2)
O1—C3	1.331 (2)	C14—C16	1.505 (3)
O2—C17	1.201 (3)	C15—C20	1.466 (3)
O3—C17	1.332 (3)	C18—C19	1.448 (5)
O3—C18	1.449 (2)	C21—C22	1.447 (4)
O4—C20	1.195 (3)	C1—H1	0.9300
O5—C20	1.335 (2)	C4—H4	0.9300
O5—C21	1.445 (3)	C5—H5	0.9300
O1—H1A	0.8200	C7—H7	0.9300
N1—C12	1.379 (2)	C8—H8	0.9300
N1—C1	1.291 (2)	C9—H9	0.9300
C1—C2	1.429 (2)	C10—H10	0.9300
C2—C3	1.396 (2)	C16—H16A	0.9600
C2—C11	1.445 (2)	C16—H16B	0.9600
C3—C4	1.413 (3)	C16—H16C	0.9600
C4—C5	1.350 (3)	C18—H18A	0.9700
C5—C6	1.414 (3)	C18—H18B	0.9700
C6—C11	1.416 (3)	C19—H19A	0.9600
C6—C7	1.412 (3)	C19—H19B	0.9600
C7—C8	1.357 (3)	C19—H19C	0.9600
C8—C9	1.389 (3)	C21—H21A	0.9700
C9—C10	1.367 (3)	C21—H21B	0.9700
C10—C11	1.412 (2)	C22—H22A	0.9600
C12—C13	1.383 (2)	C22—H22B	0.9600
C13—C17	1.478 (2)	C22—H22C	0.9600
			
S1···O5	2.7985 (16)	C14···C11^i^	3.487 (2)
S1···C2^i^	3.6620 (17)	C15···C2^i^	3.592 (3)
S1···C3^i^	3.6260 (18)	C15···C3^i^	3.581 (3)
S1···H1	2.5900	C16···O2	2.993 (3)
O1···O3	3.209 (2)	C16···O4	2.978 (3)
O1···N1	2.561 (2)	C20···C4^i^	3.523 (3)
O2···C16	2.993 (3)	C1···H10	2.6600
O3···N1	2.8195 (18)	C1···H1A	2.3900
O3···O1	3.209 (2)	C8···H18B^viii^	3.0700
O4···C16	2.978 (3)	C8···H16A^i^	3.1000
O4···C7^ii^	3.269 (3)	C9···H16A^i^	3.0700
O5···S1	2.7985 (16)	C10···H1	2.6400
O1···H19C	2.6600	C12···H1A	3.0200
O2···H18A	2.5600	C17···H16A	2.9200
O2···H21B^iii^	2.6000	C20···H16C	2.7100
O2···H9^iv^	2.6100	C20···H16B^iii^	2.9700
O2···H16A	2.8300	H1···S1	2.5900
O2···H18A^v^	2.7200	H1···C10	2.6400
O2···H16B	2.7300	H1···H10	2.0800
O2···H18B	2.6900	H1A···O3	2.5800
O3···H1A	2.5800	H1A···N1	1.8300
O3···H10^i^	2.9100	H1A···C1	2.3900
O4···H7^ii^	2.3700	H1A···C12	3.0200
O4···H21A	2.5100	H5···H7	2.4600
O4···H21B	2.7400	H7···O4^vii^	2.3700
O4···H16C	2.2500	H7···H5	2.4600
O5···H16B^iii^	2.9100	H9···O2^viii^	2.6100
N1···O1	2.561 (2)	H10···C1	2.6600
N1···O3	2.8195 (18)	H10···H1	2.0800
N1···C6^vi^	3.369 (2)	H10···O3^i^	2.9100
N1···H1A	1.8300	H16A···O2	2.8300
C1···C6^vi^	3.531 (3)	H16A···C17	2.9200
C1···C12^i^	3.319 (2)	H16A···C8^i^	3.1000
C1···C5^vi^	3.469 (3)	H16A···C9^i^	3.0700
C2···C11^vi^	3.594 (2)	H16B···O2	2.7300
C2···C15^i^	3.592 (3)	H16B···O5^iii^	2.9100
C2···S1^i^	3.6620 (17)	H16B···C20^iii^	2.9700
C2···C2^vi^	3.469 (2)	H16C···O4	2.2500
C3···C11^vi^	3.519 (3)	H16C···C20	2.7100
C3···C15^i^	3.581 (3)	H18A···O2	2.5600
C3···S1^i^	3.6260 (18)	H18A···O2^v^	2.7200
C4···C20^i^	3.523 (3)	H18B···O2	2.6900
C5···C1^vi^	3.469 (3)	H18B···C8^iv^	3.0700
C6···N1^vi^	3.369 (2)	H19B···H22B^ix^	2.6000
C6···C1^vi^	3.531 (3)	H19C···O1	2.6600
C7···O4^vii^	3.269 (3)	H21A···O4	2.5100
C11···C3^vi^	3.519 (3)	H21B···O4	2.7400
C11···C14^i^	3.487 (2)	H21B···O2^iii^	2.6000
C11···C2^vi^	3.594 (2)	H22B···H19B^x^	2.6000
C12···C1^i^	3.319 (2)		
			
C12—S1—C15	90.91 (9)	O5—C21—C22	108.0 (2)
C17—O3—C18	116.55 (16)	N1—C1—H1	119.00
C20—O5—C21	116.42 (17)	C2—C1—H1	119.00
C3—O1—H1A	109.00	C3—C4—H4	120.00
C1—N1—C12	121.62 (14)	C5—C4—H4	120.00
N1—C1—C2	122.21 (14)	C4—C5—H5	119.00
C1—C2—C11	120.78 (14)	C6—C5—H5	119.00
C3—C2—C11	119.38 (16)	C6—C7—H7	119.00
C1—C2—C3	119.82 (15)	C8—C7—H7	119.00
O1—C3—C2	123.00 (17)	C7—C8—H8	120.00
C2—C3—C4	120.20 (16)	C9—C8—H8	120.00
O1—C3—C4	116.80 (15)	C8—C9—H9	120.00
C3—C4—C5	120.63 (17)	C10—C9—H9	120.00
C4—C5—C6	121.71 (19)	C9—C10—H10	119.00
C5—C6—C11	119.22 (16)	C11—C10—H10	119.00
C7—C6—C11	119.68 (16)	C14—C16—H16A	109.00
C5—C6—C7	121.10 (18)	C14—C16—H16B	109.00
C6—C7—C8	121.1 (2)	C14—C16—H16C	110.00
C7—C8—C9	119.68 (19)	H16A—C16—H16B	109.00
C8—C9—C10	120.87 (17)	H16A—C16—H16C	110.00
C9—C10—C11	121.34 (18)	H16B—C16—H16C	109.00
C2—C11—C6	118.85 (14)	O3—C18—H18A	110.00
C6—C11—C10	117.30 (15)	O3—C18—H18B	110.00
C2—C11—C10	123.85 (16)	C19—C18—H18A	110.00
S1—C12—N1	123.46 (12)	C19—C18—H18B	110.00
S1—C12—C13	111.39 (13)	H18A—C18—H18B	108.00
N1—C12—C13	125.11 (14)	C18—C19—H19A	109.00
C12—C13—C14	113.15 (14)	C18—C19—H19B	109.00
C12—C13—C17	123.98 (17)	C18—C19—H19C	109.00
C14—C13—C17	122.84 (16)	H19A—C19—H19B	110.00
C13—C14—C16	123.87 (16)	H19A—C19—H19C	109.00
C15—C14—C16	124.86 (18)	H19B—C19—H19C	109.00
C13—C14—C15	111.23 (16)	O5—C21—H21A	110.00
S1—C15—C14	113.31 (15)	O5—C21—H21B	110.00
C14—C15—C20	127.68 (18)	C22—C21—H21A	110.00
S1—C15—C20	118.98 (14)	C22—C21—H21B	110.00
O2—C17—O3	123.34 (17)	H21A—C21—H21B	108.00
O3—C17—C13	112.87 (16)	C21—C22—H22A	109.00
O2—C17—C13	123.8 (2)	C21—C22—H22B	109.00
O3—C18—C19	109.3 (2)	C21—C22—H22C	109.00
O4—C20—O5	123.28 (19)	H22A—C22—H22B	109.00
O5—C20—C15	111.10 (18)	H22A—C22—H22C	109.00
O4—C20—C15	125.61 (19)	H22B—C22—H22C	109.00
			
C15—S1—C12—C13	0.71 (14)	C5—C6—C11—C10	178.60 (17)
C12—S1—C15—C14	−1.17 (15)	C5—C6—C7—C8	−179.4 (2)
C12—S1—C15—C20	176.74 (16)	C7—C6—C11—C2	178.44 (17)
C15—S1—C12—N1	178.59 (15)	C11—C6—C7—C8	1.0 (3)
C18—O3—C17—O2	0.1 (3)	C6—C7—C8—C9	0.7 (3)
C17—O3—C18—C19	−174.4 (3)	C7—C8—C9—C10	−1.5 (3)
C18—O3—C17—C13	179.2 (2)	C8—C9—C10—C11	0.6 (3)
C21—O5—C20—C15	−177.41 (18)	C9—C10—C11—C6	1.0 (3)
C20—O5—C21—C22	169.1 (2)	C9—C10—C11—C2	−179.21 (18)
C21—O5—C20—O4	2.0 (3)	S1—C12—C13—C14	−0.1 (2)
C12—N1—C1—C2	−177.45 (16)	N1—C12—C13—C17	0.3 (3)
C1—N1—C12—C13	178.89 (17)	N1—C12—C13—C14	−177.96 (16)
C1—N1—C12—S1	1.3 (2)	S1—C12—C13—C17	178.14 (15)
N1—C1—C2—C11	177.84 (16)	C17—C13—C14—C15	−179.04 (17)
N1—C1—C2—C3	−0.6 (3)	C12—C13—C14—C16	−178.26 (17)
C1—C2—C3—C4	178.30 (17)	C12—C13—C14—C15	−0.7 (2)
C11—C2—C3—C4	−0.2 (3)	C17—C13—C14—C16	3.4 (3)
C1—C2—C3—O1	−1.7 (3)	C14—C13—C17—O3	−143.40 (18)
C11—C2—C3—O1	179.81 (17)	C12—C13—C17—O2	−142.4 (2)
C1—C2—C11—C6	−177.67 (16)	C14—C13—C17—O2	35.7 (3)
C1—C2—C11—C10	2.6 (3)	C12—C13—C17—O3	38.5 (2)
C3—C2—C11—C10	−178.99 (17)	C13—C14—C15—C20	−176.40 (18)
C3—C2—C11—C6	0.8 (2)	C16—C14—C15—S1	178.78 (15)
O1—C3—C4—C5	180.00 (19)	C16—C14—C15—C20	1.1 (3)
C2—C3—C4—C5	0.0 (3)	C13—C14—C15—S1	1.3 (2)
C3—C4—C5—C6	−0.4 (3)	C14—C15—C20—O5	173.23 (19)
C4—C5—C6—C11	1.0 (3)	S1—C15—C20—O4	176.31 (18)
C4—C5—C6—C7	−178.60 (19)	S1—C15—C20—O5	−4.4 (2)
C5—C6—C11—C2	−1.2 (3)	C14—C15—C20—O4	−6.1 (3)
C7—C6—C11—C10	−1.8 (3)		

Symmetry codes: (i) −*x*+1, −*y*+1, −*z*+1; (ii) *x*+1, *y*+1, *z*; (iii) −*x*+1, −*y*+2, −*z*+1; (iv) *x*, *y*, *z*+1; (v) −*x*+1, −*y*+1, −*z*+2; (vi) −*x*, −*y*+1, −*z*+1; (vii) *x*−1, *y*−1, *z*; (viii) *x*, *y*, *z*−1; (ix) *x*, *y*−1, *z*+1; (x) *x*, *y*+1, *z*−1.

### Hydrogen-bond geometry (Å, °)

**Table:**

*D*—H···*A*	*D*—H	H···*A*	*D*···*A*	*D*—H···*A*
O1—H1A···O3	0.82	2.58	3.209 (2)	135
O1—H1A···N1	0.82	1.83	2.561 (2)	147
C1—H1···S1	0.93	2.59	3.0263 (18)	109
C7—H7···O4^vii^	0.93	2.37	3.269 (3)	163
C16—H16C···O4	0.96	2.25	2.978 (3)	132
C21—H21B···O2^iii^	0.97	2.60	3.565 (3)	175

Symmetry codes: (vii) *x*−1, *y*−1, *z*; (iii) −*x*+1, −*y*+2, −*z*+1.
